# The effect of early confirmation of hearing loss on the behaviour in middle childhood of children with bilateral hearing impairment

**DOI:** 10.1111/j.1469-8749.2010.03875.x

**Published:** 2011-03

**Authors:** Johannes Fellinger

**Affiliations:** Health Centre for the Deaf, Hospital of St. John of GodLinz, Austria

The hope for ‘normalization’ of the next generation of children with hearing impairments receives a new boost whenever new technology such as cochlear implants or new concepts in identification, intervention, and education are introduced into the field of deafness. These boosts run the risk of producing unrealistic expectations for the future, and neglecting the needs of the present generation of people with hearing impairments.

By showing that behaviour problems of children with hearing impairments did not improve with early confirmation of hearing loss, Stevenson et al.^1^ draw our attention to the complexity of mental health needs of those with impaired hearing, including the post-Universal Neonatal Hearing Screening (UNHS) generation.

In addition, their findings of the relatively small improvement in the receptive language ability of those confirmed before 9 months of age highlight the fact that congenital hearing loss continues to have devastating consequences.

In line with existing research, the authors showed in a previous paper^2^ that the level of behaviour problems was highest amongst those children with hearing loss with the least developed language capabilities. They follow that link in raising the question what degree of normalization of receptive language scores would be needed to eradicate the risk of increased behaviour problems in children with permanent childhood hearing impairment (PCHI) compared with hearing children.

But given that children with PCHI achieve almost normalized receptive language scores, and even including all the biological and social factors which influence mental health in general, it still leaves us with the question whether a reduced ability to hear itself contributes significantly to mental health problems observed in those with impaired hearing.

This idea is supported by findings that the level of language skills was highly correlated with the degree of hearing loss but mental health problems, especially with peer relationships, were not.^3^

The above leads us to ask the crucial question: what kind of evidence-based interventions need to follow UNHS in order to support families to actively foster the development of a strong identity and positive mental health of their child with PCHI, beyond the drive for ‘normalization’.
